# The impact of treating parental bipolar disorder and schizophrenia on their children’s mental health and wellbeing: an empty systematic review

**DOI:** 10.3389/fpsyt.2024.1425519

**Published:** 2024-08-13

**Authors:** Beril Can, Victoria Piskun, Abby Dunn, Sam Cartwright-Hatton

**Affiliations:** ^1^ School of Psychology, University of Sussex, Falmer, Brighton, United Kingdom; ^2^ School of Psychology, University of Surrey, Guilford, Surrey, United Kingdom

**Keywords:** schizophrenia, bipolar disorder, intergenerational transmission, parents, children, treatment, prevention

## Abstract

**Background:**

Parental psychosis (bipolar disorder and schizophrenia) are major risk factors for mental health problems in offspring. Although interventions that focus on parenting and the family environment have shown effectiveness in mitigating this risk, no systematic review has examined the impact of simply treating adult bipolar disorder or schizophrenia on their dependent children’s outcomes.

**Aims:**

To systematically review the effects (in randomized controlled trials) of adult-based interventions for bipolar disorder and schizophrenia, on offspring mental health and wellbeing.

**Method:**

Eligibility criteria included randomized controlled trials that examined the treatment of adults with bipolar disorder and schizophrenia that also included child mental health and wellbeing outcomes. PubMed, Scopus, PsycINFO, and PsychArticles databases were searched.

**Results:**

168,317 studies were reviewed; however, zero studies that met the inclusion criteria could be found.

**Conclusions:**

The existing research aimed at treating adult bipolar disorder and schizophrenia has so far overlooked the potential advantages that these treatments could provide for their offspring. This is a missed opportunity to understand the mechanisms of intergenerational transmission. Researchers examining treatments for adults with bipolar disorder and schizophrenia should, where appropriate, consider including both adult and child mental health outcomes in their trials.

**Systematic review registration:**

https://www.crd.york.ac.uk/prospero/display_record.php?RecordID=431007, identifier CRD42023431007.

## 1 Introduction

Bipolar disorder (BD) and schizophrenia (SZ) (often referred to as ‘psychosis’) are chronic and often incapacitating conditions. In 2019, 39.5 million individuals were affected by BD, and in 2022, 24 million individuals were affected by SZ worldwide (1, 2). The typical age of onset of BD and SZ is between late adolescence and early adulthood (3–6) at a time when many individuals start families.

It is well established that BD and SZ run in families (7–11). That is, children of parents with BD or SZ are at a high risk of developing the disorders themselves. A meta-analysis reported that the offspring of SZ parents were at a 7.54-fold risk of developing SZ, and the offspring of BD parents were at a 4.06-fold risk of developing BD themselves (9). Moreover, a large Danish study involving a cohort of 2.7 million individuals found that when one parent has SZ, offspring have a 7% risk of developing the disorder, which escalates to 27.3% if both parents are affected (12). Similarly, for BD, the risk for offspring is 4.4% with one affected parent, rising to 24.9% when both parents have BD (12). Not only do such offspring run a high risk of developing BD or SZ, but they also have an increased risk of developing other difficulties, including attention-deficit/hyperactivity disorder (ADHD), anxiety disorder, conduct disorder, behavioral and language issues during childhood, in comparison to children of parents without the disorders (13–16).

### 1.1 The intergenerational transmission of bipolar disorder and schizophrenia

Genetic, environmental, and psychosocial factors are implicated in the intergenerational transmission of BD and SZ, with genetic factors explaining a substantial proportion of the variance in comparison to other psychological disorders (17–21).

Although BD and SZ have substantial genetic heritability, environmental and psychological variables can add to this risk (9, 22–26). One environmental factor that is likely to be implicated in the intergenerational transmission of BD and SZ is parenting. Research has shown that parents with BD and SZ are likely to report disturbances associated with their parenting practices (27, 28). Research shows that such parents are more likely to have difficulties with discipline and control, dependency on the child, boundary setting, parent-child bonding, and experience higher levels of parenting stress (29–39). Difficulties in providing consistent, high-quality parenting are associated with increased mental health problems in children and may provide a partial explanation for the increased risk of mental health problems in the children of parents diagnosed with BD or SZ (36, 40–42).

### 1.2 Interventions for bipolar disorder and schizophrenia

Pharmacotherapy is effective in treating adults with BD and SZ. For instance, a meta-analysis demonstrated that adults with BD who were treated with valproate for their depressive symptoms showed a 39.3% response and 40.6% remission rate (43). In the case of SZ, the OPTIMISE study (44) found that when adults with SZ were administered amisulpride for four weeks, the remission rate was 67% of those who completed the trial.

Psychotherapy is also standard in the treatment of BD and SZ. One of the established psychosocial treatments for BD is family-focused therapy (FFT). FFT integrates psychoeducational sessions with communication and problem-solving training (45). Research on FFT has demonstrated its efficacy in mitigating depressive symptoms, reducing the likelihood of relapse, and improving psychosocial and family functioning (45–49). One line of evidence for psychosocial interventions for SZ comes from multifamily group therapy (MFGT), which combines psychoeducation, relapse prevention, social skills and occupational development, and problem-solving sessions (50). Research shows that MFGT leads to reduced stress, reduced levels of relapse and rehospitalization, and enhanced negative symptoms in adults (51–54).

### 1.3 The present study

It is clear that interventions for BD and SZ are at least somewhat effective. Given that these disorders often run in families, it would be interesting to know whether successful treatment of an adult mitigates this risk to their children. Answering this question has both clinical and theoretical implications: If treating the parent does reduce risk to the child, then CAMHS clinicians would be well-advised to partner with adult mental health services when considering the needs of children whose parents have BD or SZ. Similarly, understanding the impact of parental treatment would allow theoretical advances in our understanding of the intergenerational transmission of poor mental health. A large body of research exploring the impact of treating adult BD and SZ now exists, and this study set out to explore what this research tells us about the impact of treating adults on their children. In other words, what are the implications for children’s outcomes when their parent is treated for BD or SZ? To date, no systematic reviews have explored this question.

Thus, the present study aimed to systematically review all the RCTs investigating the impact of adult-focused BD and/or SZ interventions on their offspring’s mental health and wellbeing.

## 2 Methods

This systematic review adhered to the reporting standards outlined in the Preferred Reporting Items for Systematic Reviews and Meta-Analyses (PRISMA) guidelines (55). This review was prospectively registered with PROSPERO (registration: CRD42023431007).

### 2.1 Eligibility criteria

This study set out to identify all randomized controlled trials (RCTs) addressing interventions for adults with psychosis (BD and SZ). Studies were required to fulfill each of the following eligibility criteria for inclusion in the review.

1. The study was of adults (aged 18-65) of any gender who had or have had a diagnosis of psychosis, more specifically, either bipolar disorder or schizophrenia.
2. The study included adults as the primary participants based on their diagnosis (bipolar disorder or schizophrenia), rather than being recruited based on their child’s diagnosis.
3. The study was primary research and was published in a peer-reviewed journal either in English or Turkish.
4. Study participants were treated for adult psychosis (bipolar disorder or schizophrenia) with any type of treatment (e.g., psychological, pharmaceutical, holistic interventions). Treatment could be provided in any format (individual, group, face-to-face, online, self-help, etc.) and any setting (hospital, community center, home, etc.). There were no restrictions on the number of sessions, length, or follow-up.
5. The study compared the intervention to a control group (any other intervention such as psychological, pharmacological, holistic, treatment as usual, placebo, or to a waiting list control).
6. The study examined any mental health or well-being outcome in offspring children (under the age of 18) after the index parent’s treatment for bipolar disorder or schizophrenia.
7. The study reported the results of a randomized controlled trial.

The following were the exclusion criteria:

1. Unpublished theses, review papers, meta-analyses, prevention studies, and other grey literature.
2. Studies where the intervention’s primary aim was not treatment of adult bipolar disorder or schizophrenia.
3. Studies where the intervention included elements targeting parenting or parent-child interaction.
4. Studies where, as part of the research design, the children of those who took part in the study received treatment.

### 2.2 Information sources

Searches were conducted up to 17^th^ August 2023 with PubMed, Scopus, PsycINFO, and PsychArticles databases. To identify any other pertinent RCTs, reference lists of relevant articles, systematic reviews, and meta-analyses were also searched, and a hand search of the Bipolar Disorder Research Network was performed.

### 2.3 Search strategy

The search strategy had the objective of finding all the RCTs that investigated any treatments for adults with bipolar disorder or schizophrenia. Where possible, in each database, some search filters and limits such as “English and Turkish language”, “peer-reviewed”, and “not animal” were applied. Search terms were informed and identified by using Cochrane Library’s list of terms for bipolar disorder and schizophrenia, and Cochrane’s search strategy for identifying RCTs was used. The full search strategy for the four databases was as follows:

PubMed, PsycINFO, and PsychArticles:

[(MJMAINSUBJECT.EXACT(“Treatment Outcomes”) OR MJMAINSUBJECT.EXACT(“Placebo”) OR MJMAINSUBJECT.EXACT(“Treatment Effectiveness Evaluation”) OR MAINSUBJECT.EXACT(“Followup Studies”)] OR tiab(placebo* OR random* OR “comparative studies” OR “comparative study”) OR tiab[(“clinical trial” OR “clinical trials”)] OR tiab(“research design”) OR tiab [stud* NEAR/3 (prospectiv* OR evaluat*)] OR tiab [(singl* OR doubl* OR trebl* OR tripl*) NEAR/3 (blind* OR mask*)].

AND tiab[“Manic” OR “Manic Disorder*” OR “bipolar disorder” OR “bipolar disorders” OR “Bipolar Mood” OR “Manic Depressive” OR “Mood Disorder” OR “Bipolar Affective” OR “Bipolar” OR “Bipolar Affective Psychosis” OR “Manic-Depressive Psychosis” OR “Bipolar Depression” OR “Manic Depression” OR “Bipolar Disorder Type 1” OR “Type 1 Bipolar Disorder” OR “Type 2 Bipolar Disorder” OR “Bipolar Disorder Type 2” OR “schizophreniform disorder” OR “Schizophreniform” OR “Brief Reactive” OR “Brief Reactive Psychos*” OR “reactive psychosis” OR “Schizoaffective” OR “schizoaffective disorder” OR “Schizophrenia” OR “Psychosis”)].

Scopus:

[TITLE-ABS-KEY (“Treatment Outcomes” OR placebo OR “Treatment Effectiveness Evaluation” OR “Followup Studies” OR random* OR “comparative stud*”)] OR [(TITLE-ABS-KEY (“clinical trial*”) OR TITLE-ABS-KEY (“research design”) OR TITLE-ABS-KEY (stud* W/3 prospectiv* OR evaluat*) OR TITLE-ABS-KEY (singl* OR doubl* OR trebl* OR tripl* W/3 blind* OR mask*)].

AND [TITLE-ABS-KEY (manic OR “Manic Disorder*” OR “bipolar disorder” OR “bipolar disorders” OR “Bipolar Mood” OR “Manic Depressive” OR “Mood Disorder” OR “Bipolar Affective” OR bipolar OR “Bipolar Affective Psychosis” OR “Manic-Depressive Psychosis” OR “Bipolar Depression” OR “Manic Depression” OR “Bipolar Disorder Type 1” OR “Type 1 Bipolar Disorder” OR “Type 2 Bipolar Disorder” OR “Bipolar Disorder Type 2” OR “schizophreniform disorder” OR schizophreniform OR “Brief Reactive” OR “Brief Reactive Psychos*” OR “reactive psychosis” OR schizoaffective OR “schizoaffective disorder” OR schizophrenia OR psychosis)].

### 2.4 Study selection

The screening of studies was conducted by two independent reviewers (B.C., V.P.) using Eppi-Reviewer software. First, each reviewer screened titles and abstracts against the inclusion and exclusion criteria. A random sample of 10% of papers were double-screened by both reviewers. The level of agreement in title and abstract double-screening was 97.6% (κ = 0.73). Any disagreements in the screening on the title and abstract stage were resolved by discussion to reach a consensus. After screening titles and abstracts, papers selected for full-text screening were retrieved as far as possible.

Two independent reviewers then conducted full-text screening (B.C., V.P.). In the full-text screening, reviewers scanned the methods section of each paper to identify any outcome measures relating to children. As an additional measure, they also electronically searched the whole paper for the following terms: “offspring”, “baby”, “infant”, “child”, “adolescent”, “youth”, “parent”, “mother”, and “father”. The reviewers double-screened a random sample of 10% of the full-text articles to check for inter-rater reliability. The full-text double-screening phase returned a 100% agreement rate between the reviewers.

## 3 Results

Initially, 168,317 studies were identified from the databases and 79,886 remained after removing the duplicates (n = 88,431). After title and abstract screening, a total of 76,899 did not meet our inclusion criteria and, therefore, were excluded.

A total of 2,987 studies were sought for full-text analysis; however, 34 were unavailable. To access the unavailable papers, corresponding authors were contacted via email requesting the papers, which yielded 0 papers. Thus, 2,953 full-text papers were screened. This number comprised 2,076 full-text papers on schizophrenia, 656 on bipolar disorder, and 221 including both conditions.

**Figure 1:**
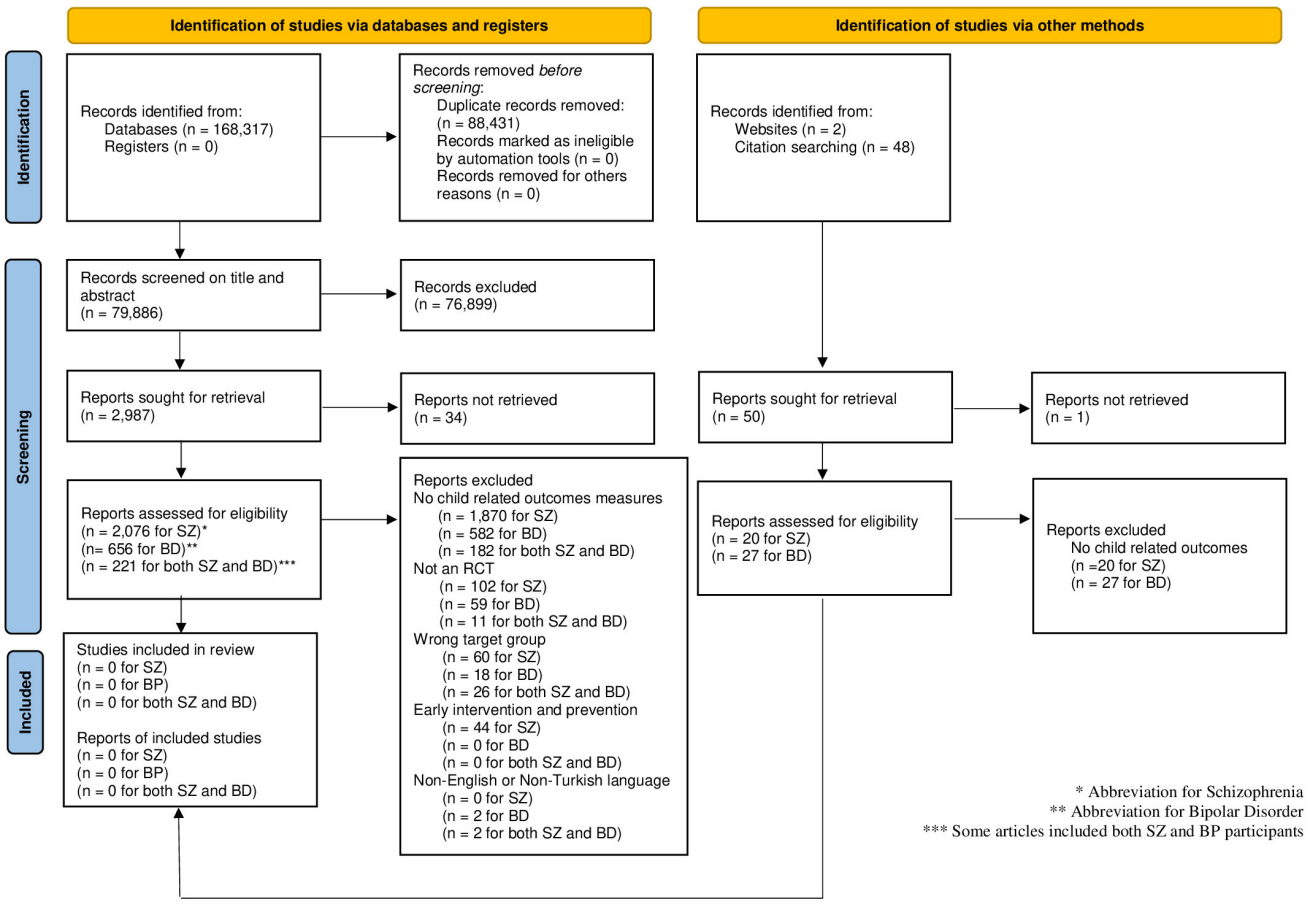
PRISMA flow diagram. Adapted from 'The Prisma 2020 statement: An updated guideline for reporting systematic reviews' by Page et al., licensed under CC-BY (55).

Out of these 2,953 full-text screened papers, zero studies met all of the inclusion and exclusion criteria. The selection process is reported in **Figure 1**, with reasons for exclusion.

## 4 Discussion

The objective of this systematic review was to determine the impact of simply treating parental BP and SZ on the mental health and wellbeing of their children. This study aimed to identify all RCTs that examined this question. We were able to find no studies that met our criteria. It should be noted that other non-RCT literature might be available and would not have been identified in this study.

The scarcity of research that matched our rigorous criteria suggests an important clinical and theoretical gap in the BD and SZ literature. Considering the well-established association between parental BD or SZ and the emergence of these disorders in children and adolescents (9, 56), this is a significant gap. Understanding the effects of treating parental BD or SZ on their children has the potential to enhance our understanding of the mechanisms that underpin the transmission of these disorders within families and to reduce the risk of poor mental health in this vulnerable set of children.

Despite a lack of papers that fully matched the inclusion criteria, some studies of BD were found that might provide some insight. We found three studies that treated adults for their BD and also looked at the impact on family functioning (57–59). These studies could not be included in the present review because they did not assess child outcomes, however, their results are summarised below.

In Miklowitz et al.’s (57) study, adults with BD were randomized to receive intensive psychosocial or collaborative care treatment. Before and after the intervention, participants were interviewed using the Longitudinal Interval Follow-up Evaluation-Range of Impaired Functioning Tool (LIFE-RIFT), which assessed their functioning across several domains, including their relationships with their children. Participants receiving intensive psychosocial treatment improved their overall LIFE-RIFT scores, as well as their relationship (with family, children or friends) scores more than participants in the control treatment (57) indicating that treating the parental BD may have had some impact on family functioning and possibly, therefore, on children’s wellbeing, although this was not directly measured. Similarly, Sylvia et al. (58) administered lithium therapy plus optimal personalized treatment (OPT) or OPT alone in adults with BD for six months. The participants also completed the LIFE-RIFT measure before and after treatment, again showing improvement in family functioning for both arms (58). Lastly, Fiorillo et al. (59) reported the results of a psychoeducational family intervention conducted with Italian adults with BD. Adults with BD were assessed on the Disability Assessment Schedule (DAS) to examine their social and personal functioning, including parental role. The participants had significant improvements in their overall DAS scores (59), suggesting that they may have experienced improvements in their parental role, although this is not entirely clear from the data that are available. Overall, the findings of these three studies suggest that addressing parental BD through adult-focused treatments could potentially lead to improvements in parent-child relationships and parenting, which, it is hoped may subsequently influence child outcomes.

Due to these studies’ limited scope in assessing actual child outcomes, it is hard to draw any firm conclusions about the impact of these interventions on child wellbeing. However, the approach of assessing these outcomes by administering a measure to the participants should be applauded. In a similar systematic review conducted by Chapman et al. (60), the aim was to assess the impact of treating parental anxiety disorder on children. However, the researchers encountered similar challenges in finding any robust data to support their investigation.

### 4.1 Implications and future research

Including a short outcome measure relating to children’s wellbeing in RCTs of adult treatments would provide basic information on whether children might also benefit from their parents’ treatment. Adding in measures assessing putative mediating mechanisms would provide information that would be of great value to those seeking to understand the role of parents in the intergenerational transmission of these conditions. Implementing this approach would not require a significant allocation of new resources. It is strongly recommended that, in the future, researchers include brief measures of children’s outcomes (and potentially some exploring mechanisms) in RCTs investigating treatments for BD and SZ, whether they are psychological or pharmacological. It is recognized that not all patients in an individual trial will have children, and that such investigations, viewed individually, would likely be underpowered. However, in combination, the results could begin to cast light on the pressing theoretical and clinical issues that concern this paper. It is also important that any researchers employing this approach carefully consider the ethical issues involved, and design their approach with researchers experienced in working with parents, and with parents who have lived-experience.

### 4.2 Strengths and limitations

This systematic review has several strengths, including an exhaustive search strategy and broad inclusion criteria: The search included a wide range of treatment modalities, including psychological, pharmaceutical, and holistic interventions, rather than being limited to a single approach. The comprehensive nature of this systematic review suggests that it is unlikely to have overlooked any relevant RCTs. The present study also demonstrated a significant level of inter-rater reliability in both the double-screening of titles and abstracts and the double-screening of full-text papers, indicating that the review process was robust.

Nevertheless, it is necessary to consider some limitations. The study sought only to include RCTs since these are considered the gold standard for investigating the effects of interventions (61). However, other research methodologies may have explored the impact of treating parental BD and SZ on children’s well-being and mental health, which were not included in this analysis. Additionally, the scope of the present study was limited to peer-reviewed studies published in either English or Turkish. This decision was made since the reviewers had fluency only in these languages and also owing to constraints in terms of time and expenses associated with translation. However, it is plausible that studies conducted in languages might have met the eligibility criteria.

## 5 Conclusion

A significant proportion of individuals diagnosed with BD and SZ also have children, and we know that these children are at increased risk of these and other disorders. It is essential to ascertain the impact of treating adults on the outcomes of children for whom they might be responsible. This review showed that the current understanding of this question is extremely limited. This gap in the literature is of significant importance given the elevated risk of transmission and the prevalence rates of BD and SZ. Addressing this gap may be accomplished without substantial financial outlay by including child-related outcome measures in existing trials of adult treatments. Collaboration among researchers, funders, and stakeholders may address this question and provide novel and valuable information for those caring for this vulnerable group of children.
